# Trends in Viral Respiratory Infections During COVID-19 Pandemic, South Korea

**DOI:** 10.3201/eid2706.210135

**Published:** 2021-06

**Authors:** Sujin Yum, Kwan Hong, Sangho Sohn, Jeehyun Kim, Byung Chul Chun

**Affiliations:** Korea University, Seoul, South Korea

**Keywords:** COVID-19, acute respiratory infections, rhinoviruses, bocaviruses, adenoviruses, influenza, human coronaviruses, respiratory syncytial virus, laboratory surveillance, SARS-CoV-2, respiratory infections, severe acute respiratory syndrome coronavirus 2, 2019 novel coronavirus disease, coronavirus disease, zoonoses, viruses, coronaviruses, South Korea

## Abstract

We compared weekly positivity rates of 8 respiratory viruses in South Korea during 2010–2019 and 2020. The overall mean positivity rate for these viruses decreased from 54.7% in 2010–2019 to 39.1% in 2020. Pandemic control measures might have reduced the incidence of many, but not all, viral respiratory infections.

The government of South Korea has implemented various measures to respond to the coronavirus disease (COVID-19) pandemic since January 20, 2020, when a case was officially reported in South Korea (*1*). Such interventions can affect the incidence of not only COVID-19 but also other respiratory viruses that are preventable with hygiene practices and social distancing (*2*–*5*). For example, in South Korea the 2019–20 influenza season ended 12 weeks earlier than in 2018–19, possibly because of the adoption of personal hygiene measures and restrictions on international travel (*3*–*5*). However, many viruses can cause acute respiratory infections; it is unknown whether other viruses also might have altered incidence or test positivity rates during the COVID-19 pandemic. We examined how the weekly positivity rates for 8 major respiratory viruses differed during the 2020 COVID-19 pandemic in South Korea compared with rates for 2010–2019.

## The Study

We analyzed surveillance data from the Korea Influenza and Respiratory Viruses Surveillance System (KINRESS) established by the Korea Disease Control and Prevention Agency (Cheongju-si, South Korea). This surveillance system documents PCR results for throat and nasal swab samples from outpatients of all ages with symptoms of acute respiratory infections at 52 sentinel medical institutions throughout the country. Samples were identified at 17 regional institutes for environmental health by real-time reverse transcription PCR for 8 viruses: adenovirus, human bocavirus (HBoV), human coronavirus (HCoV), human metapneumovirus (hMPV), human rhinovirus (HRV), influenza virus, human parainfluenza virus (HPIV), and respiratory syncytial virus (RSV) (*6*). We analyzed the weekly positivity rates of all viruses except hMPV during 2010–2019; we analyzed hMPV infections during 2012–2019 because surveillance for this disease began in 2012. We did not consider the changes in testing numerators and denominators because those raw data were not available for 2014–2017. We compared the weekly positivity rates for 2020 with those of weeks 5–52 during 2010–2019 by using a paired *t*-test. We excluded the first 4 weeks of each year to reflect the timing of the identification of COVID-19 in Korea. We reviewed the weekly positivity rates to detect any patterns already existing in the previous 10 years. KINRESS does not include data on severe acute respiratory syndrome coronavirus 2, the causative agent of COVID-19; if a physician sees a patient with suspected COVID-19, he or she refers the patient to designated COVID-19 facilities. We conducted all statistical analyses using SPSS Statistics 24.0 (SPSS Inc., http://www.spss.com).

The overall mean weekly positivity rate for all 8 viruses significantly decreased from 54.7% (SD ±8.3%) during 2010–2019 to 39.1% (SD ±15.3%) in 2020 (p<0.01) (Table). The decrease was largest for influenza virus (−9.3%, 95% CI −12.7% to −5.8%), HPIV (−6.1%, 95% CI −7.5% to −4.7%), and RSV (−2.9%, 95% CI −4.4% to −1.4%). However, the positivity rate for HRV increased by 6.6% (95% CI 2.7%–10.4%) and that of HBoV increased by 1.8% (95% CI 0.2%–3.5%) in 2020. The positivity rates for adenoviruses were not significantly different.

**Table:** Mean weekly positivity rates of respiratory viruses during weeks 5–52, South Korea, 2010–2019 compared with 2020

Virus	Mean positivity (±SD), %		Difference, % (95% CI)
	2010–2019	2020	
All studied viruses	54.7 (±8.3)	39.1 (±15.3)	−15.6 (−21.0 to −10.2)
Adenovirus	7.4 (±2.1)	6.5 (±3.2)	−0.9 (−2.1 to 0.3)
Human bocavirus	2.3 (±2.3)	4.1 (±4.5)	1.8 (0.2 to 3.5)
Human coronavirus	3.4 (±2.4)	1.2 (±2.8)	−2.2 (−3.1 to −1.3)
Human metapneumovirus*	3.1 (±3.4)	0.6 (±1.4)	−2.5 (−3.5 to −1.4)
Human rhinovirus	17.4 (±4.9)	23.9 (±15.0)	6.6 (2.7 to 10.4)
Influenza virus	11.0 (±13.4)	1.7 (±6.7)	−9.3 (−12.7 to −5.8)
Human parainfluenza virus	6.2 (±4.7)	0.1 (±0.3)	−6.1 (−7.5 to −4.7)
Respiratory syncytial virus	3.8 (±4.4)	0.9 (±1.9)	−2.9 (−4.4 to −1.4)

*Surveillance began in 2012.

In 2020, the total positivity rate for all 8 viruses decreased sharply after week 5, when COVID-19 emerged in South Korea and the government introduced nonpharmaceutical interventions (Figure). The positivity rates of HCoV, hMPV, and influenza virus abruptly decreased after week 5, reaching nearly 0 by the end of 2020. Until mid-2020, the positivity rates of RSV remained unchanged from those of the past 10 years; however, in 2020 the usual late autumn–winter outbreak of RSV did not occur. In contrast, the weekly positivity rate of HBoV increased significantly after the 40th week of 2020 compared with ratges for previous years, causing a significantly higher overall HBoV positivity rate in 2020. We did not observe substantial changes in epidemic patterns of AdV and HRV in 2020, although the average positivity rate of HRV was significantly higher than in the previous 10 years.

**Figure:**
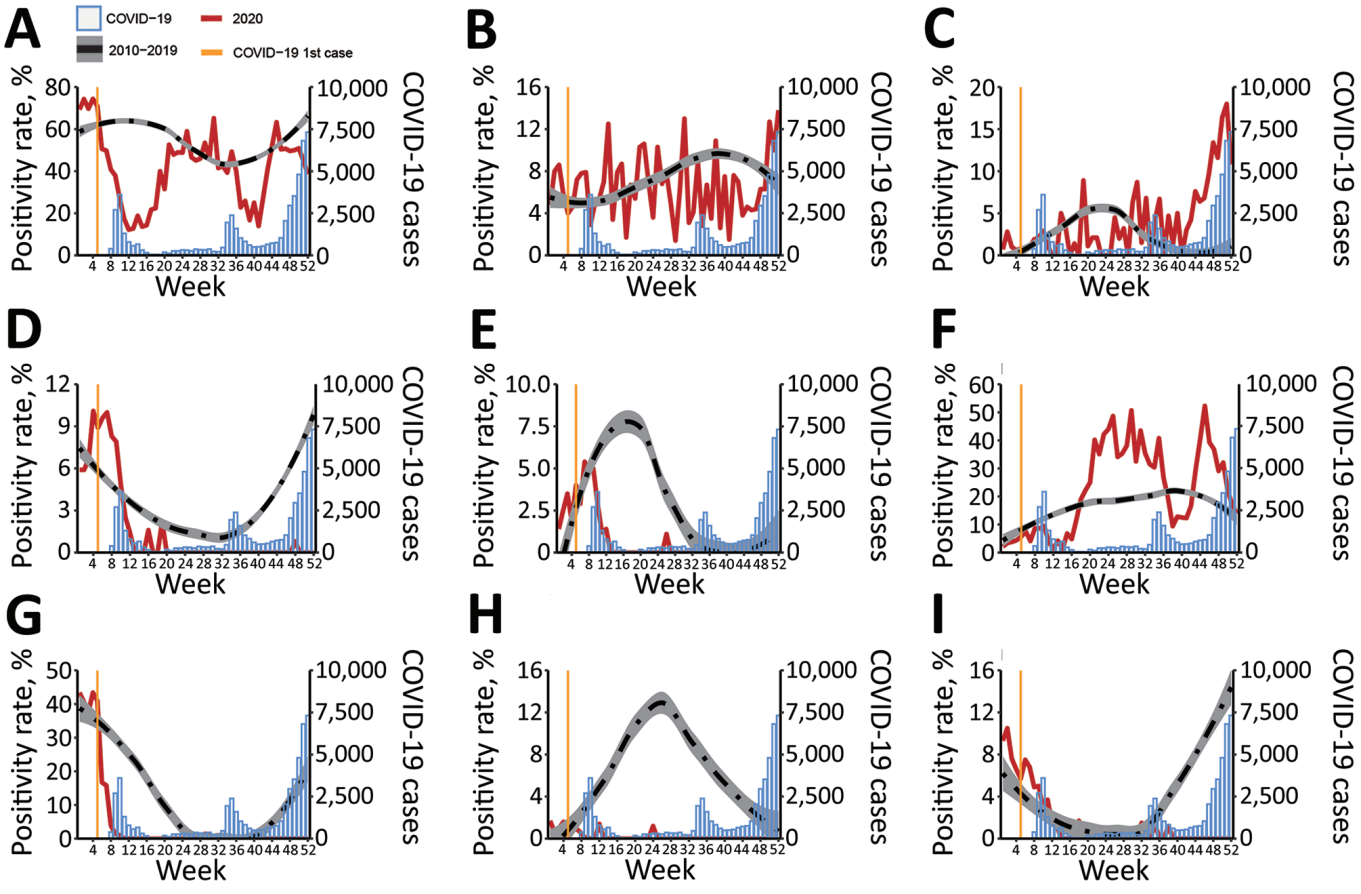
Mean weekly positivity rates for 8 respiratory viruses, South Korea, 2010–2019 compared with 2020. Vertical yellow line indicates the week of the first COVID-19 case in South Korea (i.e., the 5th week of 2020). Red line indicates weekly viral positivity rate in 2020. Blue bars show reported COVID-19 cases (Korean Ministry of Health and Welfare, http://ncov.mohw.go.kr/en). Dashed line indicates mean weekly positivity rates during 2010–2019 (data smoothed using the Loess method); gray shading indicates 95% CI. A) Total. B) Adenovirus. C) Human bocavirus. D) Human coronavirus. E) Human metapneumovirus. F) Human rhinovirus. G) Influenza virus. H) Human parainfluenza virus. I) Respiratory syncytial virus. COVID-19, coronavirus disease.

HRV and adenovirus do not have distinct seasonal trends in South Korea. KINRESS does not include data on the exact serotypes of rhinoviruses and enteroviruses. The other 6 viruses have distinct seasonalities; the timing of the peak is slightly different for each virus and changes slightly each year (*7*). HCoV, influenza virus, and RSV show peak activity in the winter (i.e., December–February); meanwhile, HBoV, hMPV, and HPIV usually show peak activity in the spring to early summer (i.e., March–June) in South Korea (Figure) (*8*). Our results suggest that the positivity rates for seasonal respiratory viruses have significantly decreased in 2020. The exception to this pattern is HBoV, which was highly prevalent in late autumn and winter of 2020, when the third surge of COVID-19 started in South Korea (Figure). The positivity rate of HBoV increased when social distancing measures were strengthened by the government. The average positivity rate of HBoV during weeks 47–52 was 13.3% (SD ±3.3%) in 2020, compared with 0.7% (SD ±0.2%) during the same period in the past 10 years. HBoV is common among children 6–24 months of age in South Korea; this infection requires treatment with oxygen and systemic steroids more frequently than other viral lower respiratory infections in children (*8*).

## Conclusions

We found that acute respiratory infections caused by seasonal viruses (except HBoV) had significantly lower positivity rates during the COVID-19 pandemic in South Korea. These overall results agree with other studies on respiratory viruses (*3*–*5*). The positivity rate of HBoV increased in November–December 2020 in proportion to the number of COVID-19 cases. The changes in the positivity rates of HRV and adenovirus, which are not seasonal, showed different patterns; positivity rates of adenovirus did not change and those of HRV significantly increased. Some studies have shown that the detection of HRV has a negative association with influenza A virus and a positive association with adenovirus, HPIV, and RSV (*9*,*10*). Little is known about the interaction of HBoV or severe acute respiratory syndrome coronavirus 2 with other respiratory viruses. Further research on the delayed outbreak of HBoV during the COVID-19 pandemic is needed.

One limitation of this study is that the changes in positivity rates do not necessarily indicate changes in incidence. The number of specimens collected during weeks 5–52 decreased to 4,576 in 2020 from 11,083 in 2019 and 10,734 in 2018. During 2014–2017, KINRESS reported only weekly positivity rates, not raw data on the numbers of specimens. It is possible that in 2020, patients with acute respiratory symptoms might have visited COVID-19 screening centers rather than other facilities. Also, most of the viruses we studied comprise multiple types and species, including many with different circulation patterns. During the study period, case detection and laboratory methods did not change for healthcare centers affiliated with KINRESS. 

In conclusion, nonpharmaceutical interventions such as social distancing might have altered trends in seasonal outbreaks of respiratory viruses during the COVID-19 pandemic in South Korea. The effects of these interventions vary for each virus. These results show that respiratory viral activities should be monitored continuously during the pandemic.
